# 24088 Investigation of the Apelingeric System on Oxidative Imbalance within Cardiorenal Syndrome Type 4

**DOI:** 10.1017/cts.2021.426

**Published:** 2021-03-30

**Authors:** Adaysha Williams, Alison Kriegel

**Affiliations:** Medical College of Wisconsin

## Abstract

ABSTRACT IMPACT: Approximately 15% of US adults have chronic kidney disease with over 700,000 of those in the end stages where treatment options are severely limited to dialysis or kidney transplant; the research presented here will help identify novel strategies that address oxidative imbalance and preserve renal function. OBJECTIVES/GOALS: Chronic Kidney Disease patients often develop secondary cardiovascular disease - Cardiorenal Syndrome Type 4. RNA sequencing data show increased apelin receptor expression in 5/6 nephrectomy rats. The Apelinergic (APJ) system is deemed beneficial in normal physiological systems. Here we explore links between stress and the APJ system in CRS4. METHODS/STUDY POPULATION: In preliminary studies performed in NRK cells, inflammatory cytokines, IL-1β and IL-6, caused increases in apelin receptor transcripts and decreased apelin transcripts, respectfully. The literature describes inflammatory processes that contribute to degradation of many organs (kidneys, heart, and liver) suggesting an oxidative imbalance. To investigate this imbalance within CRS4, three rat cell types’’ H9c2 cardiomyocytes, HII4E hepatocytes, and NRK renal epithelial cells” will be used to assess the role of exogenous apelin on pro- and anti-oxidant levels. Cells will be pretreated with apelin or vitamin E 48 hours prior to the addition of toxins or cytokines (uremic: uric acid and d-galactose or hydrogen peroxide; cytokines: IL-1β and IL-6), to assess pro- and anti-oxidant protein levels via Western Blot. RESULTS/ANTICIPATED RESULTS: We anticipate with toxin or cytokine addition (either uremic, hydrogen peroxide, IL-1β or IL-6) in all cell types, an increase in protein levels for GPX’‘ a known measure of oxidative stress’‘ should be greater than the increases in antioxidants’‘ SOD1 and Catalase. After pre-treatment of vitamin E, GPX protein levels should decrease compared to toxin/cytokine control, while SOD1 and catalase protein levels increase; this coincides with vitamin E inducing antioxidant activity in animals and humans. The anticipated results for this study after exogenous apelin addition should reveal in all three cells types reduced levels of GPX and increased levels of SOD1 and Catalase comparable to the vitamin E treatment. DISCUSSION/SIGNIFICANCE OF FINDINGS: If addition of apelin causes an antioxidant response in all three cells types, this can build on evaluation of the APJ system as a therapeutic option for those with CKD and CRS4 to minimize both inflammatory and oxidative stress. With the data gathered here, we expect to recreate the results in a CKD rat model that highlights these same manifestations.

